# How Self-Appraisal Is Mediated by the Brain

**DOI:** 10.3389/fnhum.2021.700046

**Published:** 2021-06-29

**Authors:** Gennady G. Knyazev, Alexander N. Savostyanov, Andrey V. Bocharov, Pavel D. Rudych

**Affiliations:** ^1^Laboratory of Psychophysiology of Individual Differences, Federal State Budgetary Scientific Institution Scientific Research Institute of Neurosciences and Medicine, Novosibirsk, Russia; ^2^Humanitarian Institute, Novosibirsk State University, Novosibirsk, Russia; ^3^Laboratory of Psychological Genetics at the Institute of Cytology and Genetics Siberian Branch of the Russian Academy of Sciences, Novosibirsk, Russia

**Keywords:** self-esteem, self-referential processing, trait adjective judgment task, fMRI, DCM, multilevel mediation analysis

## Abstract

Self-appraisal is a process that leads to the formation of self-esteem, which contributes to subjective well-being and mental health. Neuroimaging studies link self-esteem with the activity of the medial prefrontal cortex (MPFC), right temporoparietal junction (rTPJ), posterior cingulate cortex (PCC), anterior insula (AIns), and dorsolateral prefrontal cortex. It is not known, however, how the process of self-appraisal itself is mediated by the brain and how different nodes of the self-appraisal network interact with each other. In this study, we used multilevel mediation analysis of functional MRI data recorded during the trait adjective judgment task, treating the emotional valence of adjectives as the predictor, behavioral response as the dependent variable, and brain activity as the mediator. The mediation effect was revealed in the rTPJ. Dynamic causal modeling showed that positive self-descriptions trigger communication within the network, with the rTPJ exerting the strongest excitatory output and MPFC receiving the strongest excitatory input. rAIns receives the strongest inhibitory input and sends exclusively inhibitory connections to other regions pointing out to its role in the processing of negative self-descriptions. Analysis of individual differences showed that in some individuals, self-appraisal is mostly driven by the endorsement of positive self-descriptions and is accompanied by increased activation and communication between rTPJ, MPFC, and PCC. In others, self-appraisal is driven by the rejection of negative self-descriptions and is accompanied by increased activation of rAIns and inhibition of PCC and MPFC. Membership of these groups was predicted by different personality variables. This evidence uncovers different mechanisms of positive self-bias, which may contribute to different facets of self-esteem and are associated with different personality profiles.

## Introduction

The self is usually conceptualized as a multidimensional construct with at least two dimensions referring to the self as experiencing subject (first person perspective) or as an object of reflections and evaluations (third person perspective) (Legrand, 2003). From the third-person perspective, the self could be characterized in terms of desirable and undesirable qualities. The result of such self-evaluation is variously called self-esteem, self-worth, self-regard, self-respect, or self-confidence and is frequently conceptualized in terms of a trait, which describes inter-individual variability in the tendency to evaluate oneself positively rather than negatively (Baumeister, 1999). The universality of this tendency is reflected in the fact that trait self-esteem correlates moderately with the general factor of personality (GFP), implying that the tendency of seeing oneself through rose-colored glasses may underlie the residual covariance of the Big Five personality factors (Erdle et al., 2010; Erdle and Rushton, 2011; Simsek, 2012). The opposite tendency, which is low self-esteem, is a robust predictor of depression (Orth and Robins, 2013; Sowislo and Orth, 2013). Self-esteem is implicitly shaped throughout life as a result of the private self-evaluation of a person or as a perception of their acceptability to other people (MacDonald et al., 2003). It is theorized that the importance of positive self-esteem for an individual is mediated by the importance of being a valued member of a social group (Baumeister and Leary, 1995; Leary et al., 1995; Leary and Baumeister, 2000). Many philosophers and social scientists describe the need of seeing oneself in a positive light as a principal force of human behavior (James, 1890; McDougall, 1908; Becker, 1968).

Brain underpinning of positive self-bias is poorly understood. All relevant studies are mostly concerned with brain correlates of self-esteem and could be roughly divided into two categories. The first one includes studies in which self-esteem measures were correlated with neural responses to evaluative feedback from other people. It has been shown that negative feedback is accompanied by activation in several brain regions, such as the anterior cingulate cortex (ACC), medial prefrontal cortex (MPFC), and anterior insula (AIns), and this activation is significantly stronger in people with lower self-esteem (Masten et al., 2009; Onoda et al., 2010; Somerville et al., 2010; Eisenberger et al., 2011; Sebastian et al., 2011; van Harmelen et al., 2014; Bolling et al., 2015; Gonzalez et al., 2015; Rudolph et al., 2016; Will et al., 2016, 2017; Wang et al., 2017; van Schie et al., 2018; Peng et al., 2019). Studies falling in the second category investigated how self-esteem is associated with changes in brain activity during self-appraisal. In these studies, significant effects have been also found within social brain structures, such as the MPFC/ACC and rTPJ (Beer and Hughes, 2010; Miyamoto and Kikuchi, 2012; Yang et al., 2012, 2016; Frewen et al., 2013; Chavez and Heatherton, 2015; Hoefler et al., 2015; Izuma et al., 2018; Jiang et al., 2018; Li et al., 2019), and also in reward-related regions (Beer and Hughes, 2010; Chavez and Heatherton, 2015; Yang et al., 2016; Izuma et al., 2018), as well as in the dorsolateral prefrontal cortex (DLPFC) (Brühl et al., 2014; Jiang et al., 2018) and AIns (Schmitz and Johnson, 2007; Van der Meer et al., 2010; Modinos et al., 2011).

Some studies investigated the association of trait self-esteem with resting state functional connectivity and showed that it is linked to core regions in the default mode network (DMN) (Pan et al., 2016). In a study by Agroskin et al. (2014), structural MRI in conjunction with voxel-based morphometry was used to reveal the structural basis of trait self-esteem. Interestingly, positive associations between self-esteem and regional gray matter volume were found not only in social brain structures, such as the ACC and right TPJ but also in the right DLPFC involved in executive control functions (Rossi et al., 2009; Paneri and Gregoriou, 2017). This latter finding is consistent with the finding of Jiang et al. (2018) and is in line with the notion linking self-esteem with the cognitive control of negative emotion. Thus, Taylor et al. (2008) found higher DLPFC activity along with lower amygdala activity and cortisol level during a threat regulation task in high self-esteem individuals, in line with the evidence implicating the DLPFC in affect regulation (Hariri et al., 2003; Ochsner, 2006; Kanske et al., 2011).

In most of the above-described studies, self-appraisal was contrasted with the evaluation of other people. Some studies explicitly tested the difference in the association of self-esteem with brain activity related to self-appraisal vs. social feedback (e.g., Yang et al., 2016), but in this case, self-appraisal was also contrasted with the appraisal of other people. Thus, in spite of the multitude of neuroimaging studies of self-esteem, they do not allow to distinguish unambiguously brain activity related to self-appraisal from that related to the differentiation of self from other people. This is an important limitation, and analogous concerns were raised regarding the study of brain correlates of self-referential processing generally. Thus, an influential theory of self, which posits cortical midline structures (CMSs) as the seat of self in the brain (Northoff and Bermpohl, 2004; Northoff et al., 2006, 2011) has been criticized by Legrand and Ruby (2009), who point out that most evidence supporting this theory has been obtained in experiments contrasting self- and other-referential processing and reflects, therefore, a process of differentiating self and non-self, rather than self-referential processing *per se*. It should also be noted that correlating self-report measures of self-esteem with self-appraisal-related brain activity may confound the effect of positive self-bias, which is present in both measures. In this case, using self-report measures of self-esteem could be considered redundant, since the very process of self-appraisal already reflects the level of self-esteem.

Another question, which, to the best of the knowledge of the authors, has not been sufficiently addressed in the literature, concerns the brain underpinning of two facets of self-esteem bias. One may argue that self-esteem might be boosted either by endorsing positive self-descriptions or rejecting negative ones. These two ways of self-enhancement may have different manifestations in brain activity and connectivity. One may speculate, for instance, that dealing with negative self-descriptions (and rejecting them) may need greater involvement of emotion processing and emotion regulation capacities, which might be reflected in additional activation/connectivity of respective brain areas, such as the AIns and DLPFC (Schmitz and Johnson, 2007; Van der Meer et al., 2010; Brühl et al., 2014; Jiang et al., 2018).

Summing up, two questions remain unanswered in the field of self-appraisal research. First of all, the brain underpinning of self-appraisal *per se* (i.e., without contrasting it with appraisal of other people or correlating it with self-reported self-esteem measures) and, correspondingly, brain underpinning of positive self-bias has not been investigated. Second, the brain underpinning of two possible ways of self-enhancement (i.e., endorsement of positive self-descriptions and rejection of negative ones) has not been studied. In this study, we aimed to answer these questions using fMRI data recorded during the classical trait adjective judgment task (Kelley et al., 2002; Heatherton et al., 2006). We proceeded from the assumption that the association between positive vs. negative valence of the stimulus and its endorsement vs. rejection would reflect the level of positive self-bias, and that this association would be mediated by brain activity. To this end, we used the multilevel mediation analysis of fMRI data (Wager et al., 2008) recorded during the trait adjective judgment task, treating the manipulated parameter (i.e., the variation of the emotional valence of presented adjectives across the trials) as the predictor, affirmative vs. rejecting response as the dependent variable, and brain activity as the mediator. In such a way, we intended to avoid the use of additional redundant measures of self-esteem and to measure the self-appraisal-related brain activity directly without contrasting it with the appraisal of other people. We also expected that such an approach may additionally reveal individual differences in brain mechanisms underlying the two ways of self-esteem boosting discussed above. It could be expected, for instance, that in some individuals, brain activity in certain regions may increase upon presentation of positive descriptions, and this increase would correlate with increased probability of the affirmative response, whereas in others, brain activity may increase upon presentation of negative descriptions, which, in turn, would correlate with the increased probability of the negative response. If such individual differences are revealed, we intended to investigate their brain underpinning using the dynamic causal modeling (DCM) approach (Friston et al., 2003). In line with existing evidence, we expected to find appraisal-related effects in the MPFC/ACC, PCC, rTPJ, AIns, and DLPFC.

## Materials and Methods

### Participants

In this study, we used the data, which have already been described in the previous studies (Knyazev et al., 2020a,b) but have been reanalyzed here in a completely different way. Fifty undergraduate and postgraduate students and staff members participated in the study. All the participants received a monetary reward for their participation. Exclusion criteria were major medical illness, history of seizures or substance abuse, and all contraindications against MRI. Three participants were excluded because of fMRI artifacts, leaving 47 subjects (26 females, mean age 23.5, SD 4.9, all right-handed). The study conforms with the World Medical Association Declaration of Helsinki and was approved by the Scientific Research Institute of Neurosciences and Medicine ethical committee. All the participants gave written informed consent.

### Stimuli and Task

We used the well-known trait adjective judgment task (Kelley et al., 2002; Heatherton et al., 2006). For this task, a list of 150 adjectives was initially generated. Most words were taken from personality questionnaires, others from descriptions of appearance. A frequency dictionary of the modern Russian language (Sharoff et al., 2013) was used to estimate the frequency of word distribution. Thirty-five experts (lecturers and students from the humanitarian department of Novosibirsk State University) rated each adjective using an 11-point scale (from −5 to +5) on the desirability of respective traits. Intra-class correlation analysis using two-way mixed effects model showed high level of agreement between raters (ICC = 0.94, *p* < 0.001). Based on the average rating, 25 positive (ratings from 3 to 5), 25 neutral (ratings from −2 to 2), and 25 negative (ratings from −5 to −3) adjectives were selected. On average, they did not differ in length and the number of vowels and the frequency of word distribution. The experiment was performed using the Inquisit 6 Lab software (Millisecond Software, Seattle). The adjectives were presented visually using a rectangular projection screen with a mirror positioned within the head-coil and were presented in black color at the center of a gray screen.

The procedure consisted of four conditions, labeled “Me,” “Friend,” “Stranger,” and “Enemy,” which, in different subjects, alternated pseudo-randomly. In each condition, the participants were presented with adjectives and were asked to judge whether the respective trait applied to themselves, or (in other conditions) to some other person, such as a best friend, neutral stranger, and unpleasant person. At the beginning of each trial, the pause between the upcoming fMRI frame onset and adjective presentation onset was randomly varied between 100 and 2,350 ms intervals using a near-exponential jitter to ensure the estimation of trial-specific BOLD responses (Hinrichs et al., 2000). The participants responded by pressing the left (No) or right (Yes) button using the index fingers of their left and right hand, respectively, and the adjective instantly disappeared. Next trial started 5 s after the onset of adjective presentation. Therefore, the task lasted for 75^*^5 = 375 s and included for each participant 25 positive, 25 neutral, and 25 negative adjectives. Word order was randomized, and no adjective was presented twice.

### fMRI Data Acquisition and Preprocessing

Whole brain fMRI data were acquired with an EPI sequence on a 3.0-T scanner Philips Ingenia 7FN8GDI 3.0 T, United States. The first five volumes were discarded to allow for scanner equilibration effects, leaving 225 volumes (TR 2.5 s, TE = 35 msec, flip angle = 90°, percent phase FOV = 100, 96 × 94 matrix, 25 slices of 5 mm thickness, no gap), which covered the preliminary stages and the trait adjective task itself. High-resolution 1 mm T1-weighted structural scans were acquired with a 3D MP-GR sequence (TR = 7.8 ms, TE = 3.76 ms, 252 × 227 matrix). Prior to preprocessing, global outlier time points were identified for each participant using frame-wise displacement time-series, which were calculated using ARtifact Detection Tools (ART) (https:nitrc.org/projects/artifact_detect/). Outliers were defined as volumes with a frame-wise displacement value greater than 3 standard deviations (Atlas et al., 2014) and were modeled at the first-level general linear model analysis using dummy variables together with other nuisance regressors. The mean (SD) number of regressed out time points was 11.08 (6.07). Preprocessing was performed using the SPM-12 toolbox and included slice-time correction, realignment using rigid body transformation, co-registration, and normalization to the Montreal Neurological Institute (MNI) template, resampling to 2 × 2 × 2 mm, and smoothing (full-width half-maximum, 6 mm). We checked for motion parameters, which might induce false-positive results (Van Dijk et al., 2012). The cutoff for motion quality of the images was set at 2 mm for the three translation planes and all participants who exceeded this motion threshold were excluded from the subsequent analysis (three participants were excluded from the initial sample of 50). Next, we performed the principal components analysis (PCA) for evaluation of the level of noise and potentially as a tool for noise reduction (Thomas et al., 2002; Kay et al., 2013; Atlas et al., 2014). PCA was performed on the 4D (3D + time) dataset of each subject, and 10 components were extracted. A task-related design matrix (timing of adjective presentations modeled with boxcar function and convolved with a canonical hemodynamic response of SPM plus derivatives) and a nuisance-related design matrix (time series of outlier time points and motion parameters) were regressed on each component time series, and task- and nuisance-related *R*^2^ values were used to evaluate how task- and nuisance-related each component was. In all the subjects, all task-related *R*^2^ values were >0.1, and the nuisance-to-task ratio was <2; therefore, all components were retained (Atlas et al., 2014).

### GLM Single Trial Analysis

Unlike the usual first-level GLM, which treats similar events (e.g., all stimuli of a certain category) as one factor, in mediation analysis, each trial is treated as a unique event with its own input and output characteristics. This subsequently allows to build the mediation model using the variation of input characteristics across the trials as the predictor, behavioral responses as the dependent variable, and brain activity as a mediator (Wager et al., 2008, 2009; Atlas et al., 2014). Therefore, the GLM design matrix was constructed with separate regressors for each trial. Each regressor was modeled by a boxcar function with on and off points corresponding to the time at which the adjective was presented and the time when a subject pressed the response button, respectively. Each trial was modeled using a flexible basis set that includes not only the canonical hemodynamic response (HRF) but also its time derivative. We opted not to use the dispersion derivative, which is important for modeling slow and prolonged responses, such as responses to noxious heat (e.g., Atlas et al., 2010, 2014) but should not be so important for modeling fast responses to visual stimuli, while increasing the design matrix collinearity. Since some trials might be contaminated by movement artifacts, we calculated for each subject trial-by-trial variance inflation factors (VIFs), a measure of the collinearity of each trial with nuisance regressors (i.e., estimated head movement: x, y, z, roll, pitch, and yaw). All trials with VIFs >2 were excluded from analyses (M = 0.13, SD = 0.33). Subsequently, the first-level GLM design matrix of each subject included regressors for stimulus-evoked responses for each trial, 12 head movement nuisance regressors (x, y, z, roll, pitch, yaw, and these vectors squared), the indicator vector for time points estimated as outliers (see the previous section), and the column of ones. There are different options for the choice of a summary estimate of the effect of each trial. The standard beta regressor amplitude is the most obvious choice. The area under the curve (AUC) of each trial-wise fitted response is another choice that is particularly relevant for such kind of stimuli as the noxious heat, which has been shown to influence not only amplitude but also the duration of evoked HRF (Atlas et al., 2010, 2014). We compared preliminary results using both these options and found no major differences. Therefore, results obtained using the standard beta regressor amplitude are reported for the sake of consistency with the majority of published studies.

### Mediation Analysis

**Figure 1:**
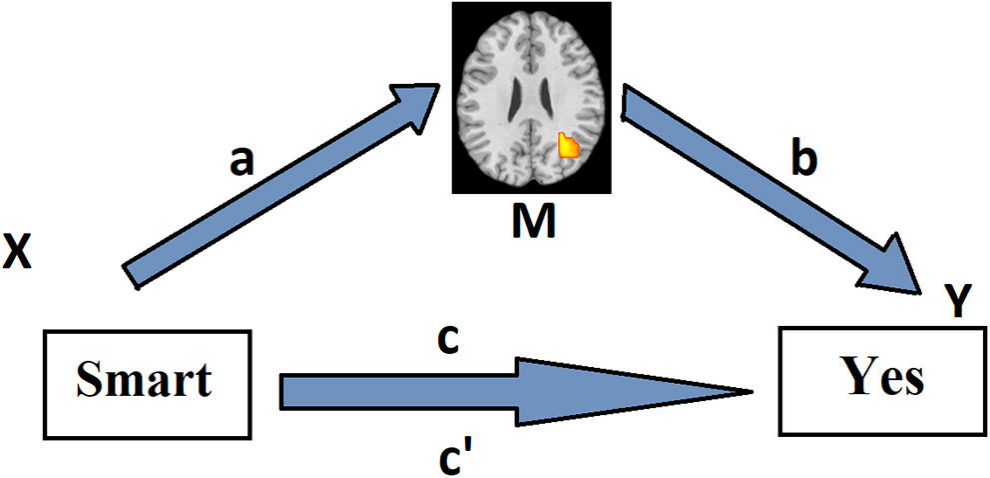
Path model for the first level of the multilevel mediation analysis. Associations between the predictor X (emotional adjective valence) and the mediator M (adjective-presentation-related brain activity) (path *a*) and between the mediator M and the dependent variable Y (behavioral choice) (path *b*) across the trials are assessed voxel-wise by means of linear regression method in each subject separately. The mediation effect, i.e., the effect of X on Y as mediated by M (path a^*^b), is calculated as the product of the resulting two regression coefficients. Path *c* reflects the total relationship between adjective valence and behavioral response across the trials in a particular subject. Path *c* reflects this relationship, controlling for activity in a brain voxel.

We used the mediation effect parametric mapping (MEPM) to examine the association between trial-by-trial variation of adjective valence (coded: negative = −1, neutral = 0, positive = +1) and the behavioral response (no = −1, yes = +1), as mediated by brain activity (Wager et al., 2008, 2009; Atlas et al., 2010). The MEPM uses the standard mediation path model (Baron and Kenny, 1986), in which a predictor X (in this case, adjective valence) is related to an outcome Y (behavioral response) and this relationship is mediated by a mediator M (brain activity) (Figure 1). At the first level, this model is fitted in each subject on a voxel-by-voxel basis. For a mediation result to be significant within a voxel, M must be related to X (path *a*), M must be related to Y after controlling for X (path *b*), and the indirect relationship (a^*^b) must also be significant. Moreover, the overall relationship between X and Y must decrease when controlling for X-evoked responses within the voxel. The significance of the mediation effect was tested using bias-corrected, accelerated bootstrap tests (10,000 samples) (Efron and Tibshirani, 1993).

At the second level, the significance of effects revealed at the first level is tested in the group of subjects. The second-level analysis also allows to reveal individual differences in brain mechanisms underlying the mediation effect. The mediation effect might be negative (i.e., *a* and *b* have opposite signs) or positive (*a* and *b* have the same sign). In the latter case, both signs might be positive or negative. Moreover, they could be positive in some subjects and negative in others. Thus, the mediation effect could be driven either by consistent effects (i.e., *a* and *b* have the same sign in all subjects) or by the covariance between *a* and *b* across individuals (i.e., they are both positive in some but both negative in others) (Kenny et al., 2003). For instance, it is possible that in some individuals a particular brain region may increase its activity upon presentation of positive adjectives and that increased activity in this brain region is associated with an increased probability of a confirmatory response. In other subjects, however, this region might increase its activity upon presentation of negative adjectives, and this is associated with an increased probability of a negative response. These individual differences may help to understand the brain underpinning of two different ways of self-esteem boosting, i.e., by endorsing positive self-descriptions or rejecting negative ones. To examine these individual differences, trial-wise data of each cluster showing significant mediation effect should be extracted, and mediation analysis should be repeated on these data. Afterwards, the consistency of the *a* and *b* coefficients and their covariance across individuals could be tested using a one-sample *t*-test and correlation, respectively (Atlas et al., 2014). If a mediator is driven by covariance rather than consistent effects, we try to reveal the underlying individual differences in effective connectivity using the Dynamic Causal Modeling (DCM). To control for false-positive results, we used the false discovery rate correction at *q* < 0.05. This corresponded to a voxel-wise threshold of *p* < 0.001 for the mediation effect, and a threshold f *p* < 0.002 for the conjunction across all three maps (*a, b*, and a^*^b). Cluster extent threshold was determined using a Monte Carlo simulation implemented in the NeuroElf's (http://neuroelf.net/) instantiation of the AlphaSim function (Forman et al., 1995). For the primary threshold of 0.001, the extent threshold was determined to be 25 voxels. Cluster-wise tests were performed on data extracted from these voxels.

### Psychometric Variables

To assess individual differences in the Big-Five personality traits, we used the IPIP 50 Big-Five Factor Markers (Goldberg, 1992; Knyazev et al., 2010). In this sample, the Cronbach's alphas were 0.8 for extraversion, 0.78 for agreeableness, 0.85 for conscientiousness, 0.89 for neuroticism, and 0.8 for intellect. Eysenckian personality facets were measured by the Eysenck Personality Profiler (Eysenck et al., 2000; Knyazev et al., 2004a). For all nine scales, alphas were >0.7. Trait Anxiety was measured by the Spielberger State Trait Anxiety Inventory (Spielberger et al., 1970; Hanin, 1989, alpha = 0.91). Aggressiveness was measured by the Buss-Perry aggression scales (Buss and Perry, 1992; Knyazev et al., 2010). We used only the anger (alpha = 0.71) and hostility (alpha = 0.75) subscales. Behavioral activation and inhibition were measured by the (1994) BIS/BAS scales of Carver and White (Knyazev et al., 2004b). Cronbach's alphas were 0.71 for BIS, 0.65 for drive, 0.76 for reward responsiveness, and 0.73 for fun-seeking. We also used as psychometric variables the “Friend” (hereafter Fscore), “Stranger” (Sscore), and “Enemy” (Escore) ratings.

### Dynamic Causal Modeling

DCM (Friston et al., 2003) was used to examine the effective connectivity between brain areas revealed by means of multilevel mediation analysis. The volumes of interest (VOIs) were selected based on the (1) results of mediation analysis, (2) existing evidence about the brain regions that are consistently activated in self-appraisal tasks, and (3) if they fell within the areas that showed an activation in the group-level GLM (peak *p* < 0.001, uncorrected, within 8-mm sphere from the peak of mediation analysis results). The GLM analysis at the first-level modeled the adjective presentation by a stick function with zero duration indicating the onset of each trial, which was convolved with the canonical HRF. The stick function was modulated by two parametric modulators (adjective valence and response). Next, followed the six realignment parameters and one constant. Data were high-pass filtered with a cutoff at 128 s, and an autoregression model of polynomial order 1 was used to account for temporally correlated residuals. Model estimation was performed using a restricted maximum likelihood (ReML) fit. After model estimation, contrast images representing the effects of adjective presentation were computed for each participant and submitted to a second-level one-sample *t*-test analysis. Seven VOIs were selected based on the criteria described above: MPFC (3, 20, 50), PCC (−5, −55, 29), rTPJ (51, −52, 35), rAIns (35, −16, 17), lDLPFC (−24, 17, 47), and left (lPG, −39, −22, 59) and right (rPG, 39, −19, 56) precentral gyri. To extract the time series for these VOIs, contrast images representing the effects of adjective presentation and the two parametric modulators, as well as an “effects of interest” F-contrast [eye (3) in Malab notation] were calculated for each subject. Next, each node was modeled as a sphere with 6 mm radius and the MNI coordinates of the center determined as the closest peak coordinates for each individual subject (within 8-mm sphere from the group level peak) that exceed a liberal statistical threshold of *p* < 0.05 uncorrected. The time series was pre-whitened, high-pass filtered, and “adjusted” to the F-contrast to remove any nuisance effects. Finally, a single representative time series was computed for each VOI by extracting the principal eigenvariate (Zeidman et al., 2019a).

We used the bilinear, deterministic, single-state DCM with mean-centered inputs, as implemented in SPM12. The adjective presentation regressor was used as the driving input, and the adjective valence parametric regressor was used as a putative modulator of effective connectivity. While setting the context-independent effective connectivity among the seven brain regions (matrix A), all but lPG and rPG VOIs were allowed to be fully interconnected. For the sake of simplicity, the lPG and rPG were allowed to be connected with each other and receive inputs from all the other regions but were not allowed to influence the other regions. Since DLPFC and AIns are the central hubs of two attention regulation networks (i.e., the central executive and the salience networks, Dosenbach et al., 2007; Seeley et al., 2007; Vincent et al., 2008), whereas MPFC, PCC, and TPJ belong to the DMN, which is mostly associated with internally oriented attention (Raichle et al., 2001; Buckner et al., 2008; Davey et al., 2016), the DLPFC and AIns were assumed to act as input regions. Preliminary analyses showed that the model, which included both these regions as input, has a clear advantage compared with models with anyone of these regions. All the connections that were specified in the matrix A, apart from lPG and rPG interconnections, were allowed to be modulated by the adjective valence. The Parametric Empirical Bayes (PEB) analysis based on Bayesian posterior inference (Friston et al., 2016) was performed to reveal group level effects. In this analysis, posterior probability (PP) is used as an indicator of the confidence in whether a modulatory change in a group is different from zero (or different compared with another group) (Friston and Penny, 2003). A very important advantage of PEB is the lack of false positives and multiple-comparison problem (Friston and Penny, 2003). Rather than testing specific hypotheses, we opted for a more exploratory approach using an automatic search of nested models, which prunes parameters from the fully connected PEB model that does not contribute to the model evidence. This is called Bayesian Model Reduction (BMR). Next, parameters (connection strengths) from the best reduced models are averaged (Bayesian Model Averaging) to produce parameter estimates (Zeidman et al., 2019b).

## Results

### Behavioral and Psychometric Results

Mean (SD) score in the trait adjective judgment task calculated as a proportion of trials in which either positive description was endorsed or negative one was rejected was 0.26 (0.17) with the maximal possible score being 0.67. Mean (SD) correlation (Fischer *Z*-transformed) between the input (adjective valence) and the output (affirmative behavioral response) (hereafter IOC) in the trait adjective judgment task was 0.37 (0.28), ranging from −0.16 to 0.87, meaning that most of the participants showed positive self-bias. The two variables strongly correlated with each other (*r* = 0.99, *p* < 0.001). Among the personality variables, IOC correlated negatively with trait anxiety (*r* = −51, *p* < 0.001), anger (*r* = −0.56, *p* < 0.001), hostility (*r* = −0.41, *p* = 0.005), inferiority (*r* = −0.44, *p* = 0.002), unhappiness (*r* = −0.31, *p* = 0.035), and neuroticism (*r* = −0.51, *p* < 0.001), and positively with conscientiousness (*r* = 0.35, *p* = 0.017).

### Multilevel Mediation Analysis

In this study, we aimed to analyze self-appraisal. However, the mediation analysis was also performed for the three other experimental conditions (i.e., “Friend,” “Stranger,” and “Enemy”). Significant group-level mediation effects were revealed only in the ‘Me’ condition.

The mediation analysis consisted of three tests (Figure 1): (1) path *a*, stimulus-related brain activity; (2) path *b*, response-related brain activity, controlling for the stimulus; (3) path a^*^b, which tests whether the brain region explains a significant amount of the covariance between emotional adjective category and behavioral response. All these effects are described below.

**Figure 2:**
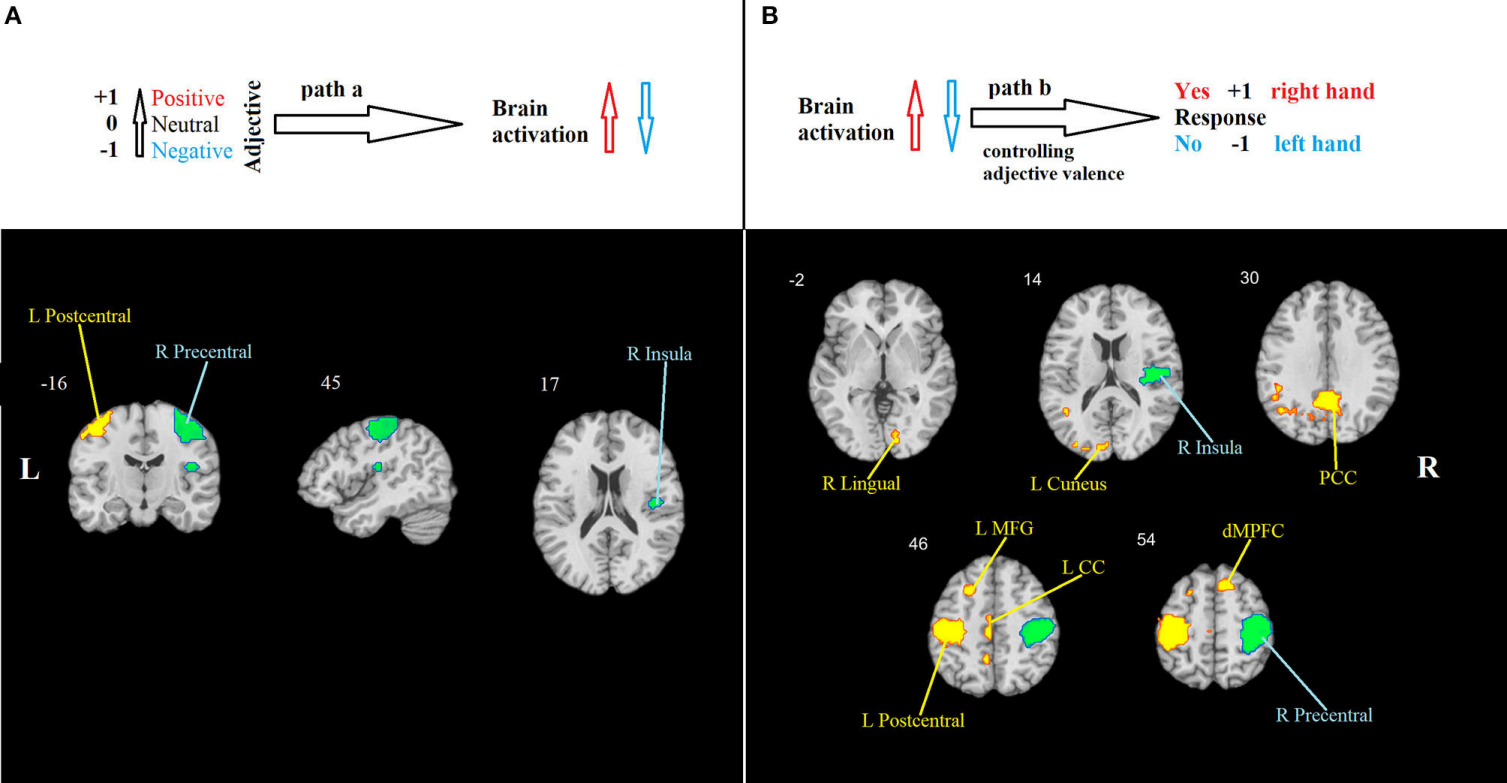
Mediation analysis results. **(A)** Path *a*: brain regions that are significantly associated with adjective valence. Hot colors show the region (the left primary somatosensory cortex) in which activity increases in response to positive valence, indicating the propensity to respond “Yes” using the right-hand button. Cool colors show regions in which activity increases in response to negative valence. They include the right primary motor cortex, indicating the propensity to respond “No” using the left-hand button, and the right insula. **(B)** Path *b*: brain regions that are significantly associated with behavioral response, controlling for adjective valence. Hot colors show regions in which activity increases when the response “Yes” is chosen, and cool colors show regions that increase their activity when the response “No” is chosen. PCC, posterior cingulate cortex; LMFG, left middle frontal gyrus; LCC, left cingulate cortex; dMPFC, dorsal medial prefrontal cortex. All effects are significant at *p* < 0.001, FWE-corrected.

Path *a*: A positive association between the desirability of the trait described by an adjective and the level of BOLD activation was found in the left postcentral gyrus. A negative association was found in the right postcentral gyrus and the right insula (Table 1, Figure 2A).

**Table 1:** Summary of the clusters that showed significant effects in the multilevel mediation analysis.

**Path**	**Direction**	**Location**	**X, Y, Z**	**BA**	**k**	***Z*_**max**_**
*a*	Positive	Left postcentral gyrus	−42, −22, 56	3	323	18.9
	Negative	Right precentral gyrus	39, −22, 56	4	554	10.0
	Negative	Right insula	35, −16, 17	13	29	8.6
*b*	Positive	Right lingual gyrus	12, −82, −4	18	29	8.9
	Positive	Left cuneus	−12, −88, 11	18	37	10.2
	Positive	Left postcentral gyrus	−39, −28, 50	2	1122	13.6
	Positive	Posterior cingulate	0, −55, 29	31	384	11.3
	Positive	Left cingulate gyrus	−3, −19, 44	24	65	10.9
	Positive	Left middle frontal gyrus	−24, 17, 47	6	35	10.3
	Positive	Medial frontal gyrus	3, 20, 50	8	53	10.7
	Negative	Right insula	35, −16, 12	13	168	10.9
	Negative	Right precentral gyrus	39, −25, 56	4	956	10.0
a*b	Positive	Right temporoparietal junction	51, −52, 35	39	35	9.1
	Positive	Right precentral gyrus	39, −19, 56	4	300	9.2
	Positive	Left precentral gyrus	−39, −22, 59	4	199	9.8
conjunction	Positive	Right precentral gyrus	39, −19, 56	4	255	8.2
	Positive	Left postcentral gyrus	−39, −22, 56	4	170	9.1

*X, Y, Z, cluster center in millimeters in MNI-space; BA, Brodmann area; k, number of voxels. Z_max_, Z-score at the peak of the cluster*.

Path *b*, i.e., behavioral response-related activity, controlling for stimulus valence, was significant in nine clusters. Positive effects were observed in the cuneus and lingual gyrus, posterior and middle cingulate gyri, left postcentral gyrus, left middle frontal gyrus, and epy right dorsal MPFC. Negative effects were found in the right insula and the right precentral gyrus (Table 1, Figure 2B).

**Figure 3:**
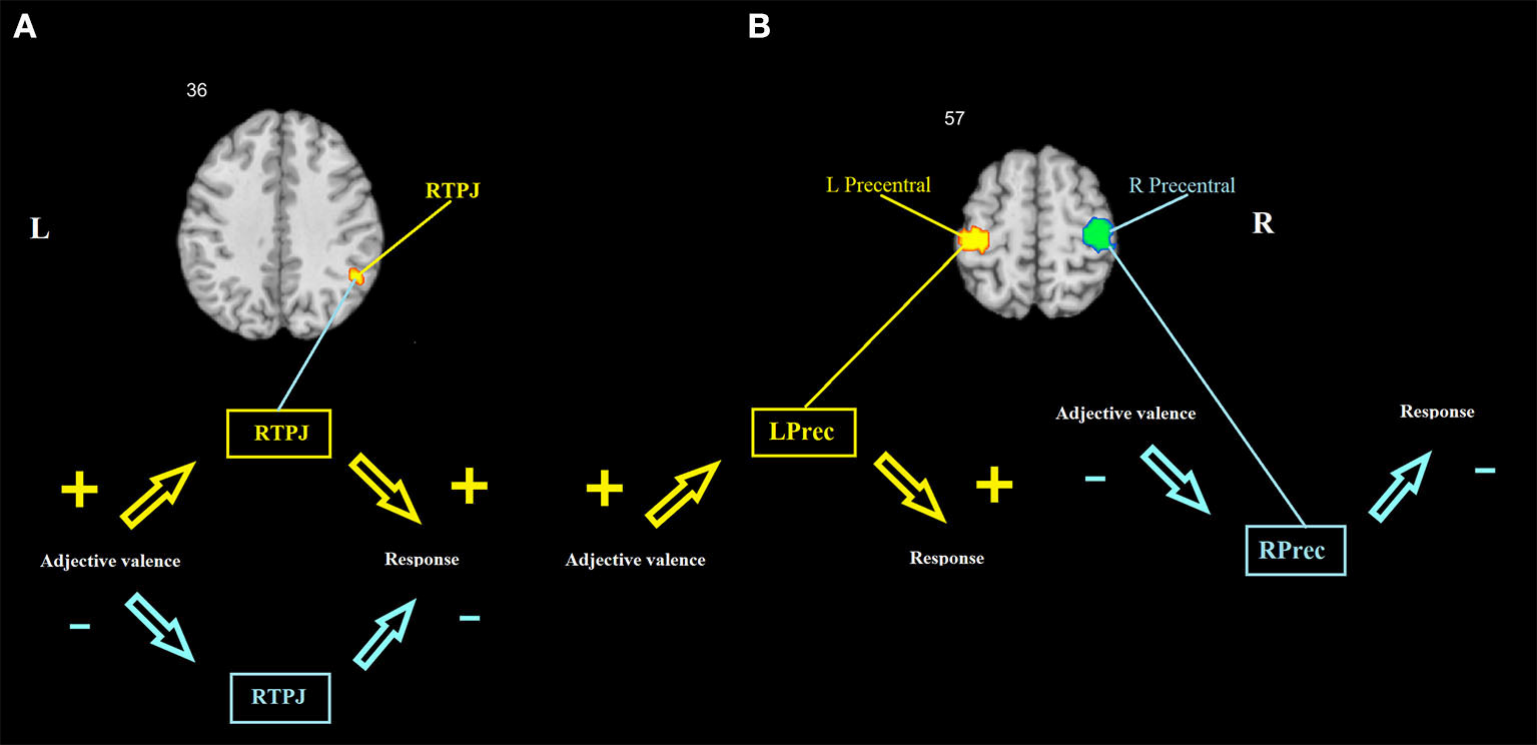
Brain mediators of the relationship between adjective valence and behavioral response. **(A)** The right TPJ shows a mediation effect that is driven by the covariance between *a* and *b* coefficients across individuals. **(B)** The left and right precentral gyri show mediation effects that are driven by consistent effects. Hot colors show a positive association between adjective valence and brain activity, and between brain activity and behavioral response. Cool colors show a negative association between adjective valence and brain activity, and between brain activity and behavioral response. RTPJ, right temporoparietal junction; LPrec and RPrec, left and right precentral gyrus.

Path *ab*: A positive mediation effect was found in the right TPJ and left (lPG) and right (rPG) precentral gyri (Table 1, Figure 3).

To test whether the mediation effects are driven by consistent effects or by the covariance between *a* and *b* across individuals, trial-wise data of each one of the three significant clusters were extracted, and the mediation analysis was repeated on these data. Afterwards, the consistency of the *a* and *b* coefficients and their covariance across individuals were tested using a one-sample *t*-test and correlation, respectively. For the left and right precentral gyri, the mediation effects were found to be consistent (for the lPG, *t* = 4.8, *p* < 0.001 and *t* = 7.03, *p* < 0.001, for the *a* and *b* coefficients, respectively, *r* = 0.01, *p* = 0.959; for the rPG, *t* = −4.7, *p* < 0.001 and *t* = −5.8, *p* < 0.001, for the *a* and *b* coefficients, respectively, *r* = 0.11, *p* = 0.466). Thus, the lPG increases its activity upon presentation of positive adjectives, and this increase is associated with increased probability of a confirmatory response; whereas the rPG, on the contrary, increases its activity upon presentation of negative adjectives, which, in turn, is associated with increased probability of a negative response. For the right TPJ, the mediation effect was found to be driven by a covariance between *a* and *b* rather than by consistent effects (*t* = −0.14, *p* = 0.884 and *t* = 1.22, *p* = 0.228, for the *a* and *b* coefficients, respectively, *r* = 0.33, *p* = 0.022). There were 17 participants who had both the *a* and *b* coefficients positive (hereafter POS) and 14 participants who had both the *a* and *b* coefficients negative (hereafter NEG). Sixteen participants had an inconsistent pattern of the *a* and *b* coefficient signs (hereafter NONE). Three dummy variables were created to match each one of these groups against all the other participants. Binary logistic regression analyses were conducted to reveal the psychological predictors of the membership of each group. In these analyses, the dummy variables described above served as dependent variables, whereas the age, gender, and all psychometric scales of the participants were used as predictors. Matching POS with all others yielded two predictors: Escore (B = −0.956, *p* = 0.01, odds ratio = 0.385) and drive (B = −0.404, *p* = 0.029, odds ratio = 0.668), with 70% of correctly classified. Matching NEG with others yielded one predictor: anger (B = −0.148, *p* = 0.044, odds ratio = 0.862), with 66% of correctly classified. Matching NONE with others also yielded one predictor: fun seeking (B = −0.335, *p* = 0.021, odds ratio = 0.716), with 77% of correctly classified.

### Dynamic Causal Modeling

**Figure 4:**
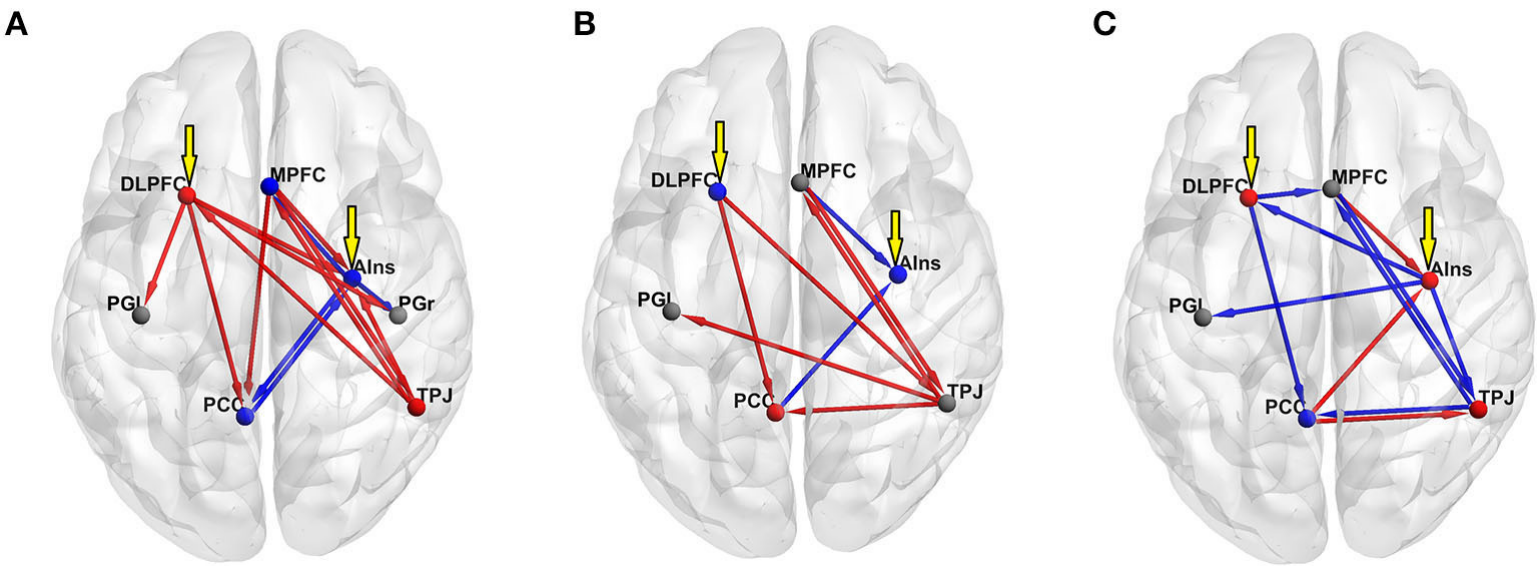
Results of the DCM PEB analyses of the effect of adjective valence on effective connectivity (all effects with “positive evidence,” i.e., PP ≥ 0.75, are shown). Red spheres show decreased self-inhibition (i.e., disinhibition) of a region, whereas blue spheres show increased self-inhibition. Gray spheres mark regions with no significant effect. Red arrows show excitatory and blue arrows show inhibitory inputs. Yellow arrows show extrinsic input to the model. **(A)** group mean; **(B)** contrasting the POS group with the rest of the sample; **(C)** contrasting the NEG group with the rest of the sample.

DCM was used to examine the effective connectivity between brain areas revealed by means of the mediation analysis. The left DLPFC and the right AIns were assumed to act as input regions, whereas lPG and rPG were considered output regions (i.e., they were allowed to receive inputs from other regions but were not allowed to influence other regions). MPFC, PCC, and TPJ (along with the left DLPFC and the right AIns) acted as modulators. The PEB was used to test the mean of each modulatory change against zero across all the participants and to test the group difference in each modulatory change using the POS, NEG, and NONE dummy variables. The age and gender of the participants were used as nuisance covariates. In the former analysis, commonalities were modeled as a column of ones, and age and gender regressors were mean-centered. The latter analyses were performed separately for each dummy variable, which was entered after the column of ones, followed by age and gender covariates. Table 2 presents all the parameter estimates (PEs) obtained by means of BMR along with their PPs. Matching the NONE group with all the others did not yield PEs with PP ≥ 0.75, which is considered a threshold for “positive evidence;” therefore, the results for this group are not presented. For each VOI, the uppermost row in Table 2 presents the results for its self-connection, which determine the sensitivity of this region to input coming from the rest of the network (Zeidman et al., 2019b). Note that positive numbers in these rows indicate increased self-inhibition, and negative numbers indicate disinhibition. All the other rows present between-region parameters, with positive and negative numbers indicating, respectively, mean increase and decrease in connectivity strength due to (in this case) increase in adjective valence, or positive and negative difference in PE between a particular group (POS or NEG) and the rest of the sample. Looking at the commonalities (group mean) and considering only the effects with “positive evidence” (i.e., PP ≥ 0.75), one may notice that the increase in adjective valence is associated with increased self-inhibition of rAIns, MPFC, PCC, and rPG, and disinhibition of rTPJ and lDLPFC. Besides, it increases an excitatory influence of rTPJ on MPFC, lDL, PFC, and rAIns and an inhibitory influence of PCC on rAIns. Finally, it increases an inhibitory influence of rAIns on rPG, which, combined with increased self-inhibition of this region, should diminish the propensity to push the “NO” button with the left hand and, correspondingly, should increase the propensity to push the “YES” button with the right hand (Figure 4A).

**Table 2:** Results of DCM PEB analyses.

	**Group mean**		**POS > others**		**NEG > others**	
**EC**	**PE**	**PP**	**PE**	**PP**	**PE**	**PP**
AIns → AIns	0.37^**^	0.99	0.82^**^	1	−0.096^*^	0.78
AIns → DLPFC	−0.028	0.71	0.002	0.51	−0.365^*^	0.94
AIns → MPFC	−0.050^*^	0.82	−0.052	0.68	0.001	0.50
AIns → PCC	−0.071^*^	0.90	−0.058	0.71	0.024	0.58
AIns → TPJ	−0.012	0.60	0.032	0.62	−0.068^*^	0.75
AIns → lPG	−0.012	0.60	0.041	0.67	−0.087^*^	0.82
AIns → rPG	−0.29^**^	1	0.030	0.62	0.002	0.51
DLPFC → DLPFC	−0.044^*^	0.78	1.033^**^	1	−1.328^**^	1
DLPFC → AIns	0.055^*^	0.85	0.037	0.63	−0.019	0.56
DLPFC → MPFC	−0.030	0.71	0.036	0.62	−0.430^**^	0.95
DLPFC → PCC	0.049^*^	0.85	0.073^*^	0.76	−0.358^**^	0.97
DLPFC → TPJ	0.010	0.58	0.078^*^	0.77	0.029	0.61
DLPFC → lPG	0.050^*^	0.85	−0.035	0.65	−0.014	0.56
DLPFC → rPG	0.050^*^	0.84	0.001	0.50	−0.08	0.74
MPFC → MPFC	0.52^**^	1	0.070	0.71	−0.058	0.68
MPFC → AIns	0.060^*^	0.87	−0.829^**^	1	0.088^*^	0.76
MPFC → DLPFC	−0.011	0.58	0.048	0.66	0.013	0.54
MPFC → PCC	0.042^*^	0.78	−0.068	0.72	0.035	0.62
MPFC → TPJ	0.035^*^	0.75	0.976^**^	1	−0.148^*^	0.89
MPFC → lPG	−0.035	0.74	−0.013	0.55	0.014	0.55
MPFC → rPG	0.015	0.62	−0.028	0.60	−0.041	0.64
PCC → PCC	0.54^**^	1	−1.281^**^	1	1.244^**^	1
PCC → AIns	−0.51^**^	1	−0.078^*^	0.75	0.406^*^	0.90
PCC → DLPFC	−0.028	0.69	−0.050	0.66	−0.028	0.59
PCC → MPFC	−0.019	0.64	−0.062	0.70	−0.032	0.61
PCC → TPJ	0.022	0.67	−0.070	0.74	0.151^*^	0.91
PCC → lPG	−0.019	0.65	0.070	0.73	−0.052	0.67
PCC → rPG	0.004	0.53	0.086	0.74	−0.08	0.74
TPJ → TPJ	−0.715^**^	1	−0.019	0.56	−0.083^*^	0.75
TPJ → AIns	0.197^*^	0.89	−0.070	0.72	−0.045	0.64
TPJ → DLPFC	0.201^*^	0.87	−0.030	0.60	0.048	0.65
TPJ → MPFC	0.604^**^	1	1.315^**^	1	−0.787^**^	1
TPJ → PCC	−0.037	0.74	0.655^**^	0.99	−0.107^*^	0.82
TPJ → lPG	0.034	0.73	0.474^**^	0.95	−0.015	0.55
TPJ → rPG	0.045	0.74	−0.033	0.61	0.034	0.61

*Posterior parameter estimates represent either the mean of the modulatory effect across subjects or the difference in modulatory effect due to group membership. For between-region parameters, positive and negative numbers indicate increase and decrease in connectivity strength, respectively. For self-connections, positive numbers indicate increased self-inhibition, and negative numbers indicate disinhibition. PP, posterior probability*.

* *PP ≥ 0.75*;

** *PP ≥ 0.95*.

To evaluate the relative importance of each node in the graph, we calculated node strengths using the *strengths_dir.m* function from the Brain Connectivity Toolbox (http://www.brain-connectivity-toolbox.net/). Node strength is the sum of weights of links connected to the node. In directed networks, the out-strength is calculated as the sum of outward link weights, and the in-strength is the sum of inward link weights. For signed networks, these measures represent a balance of excitatory and inhibitory connections. The rTPJ had the maximal positive (1.054) and the PCC had the maximal negative (−0.55) out-strength, whereas the MPFC had the maximal positive (0.505) and the rAIns had the maximal negative (−0.198) in-strength.

Contrasting the POS group with the rest of the sample yielded increased self-inhibition of lDLPFC and rAIns and disinhibition of PCC. In this group, compared with other participants, the increase in adjective valence produced higher excitatory input from rTPJ to MPFC, PCC, and lPG, and from MPFC to rTPJ, and higher inhibitory input from MPFC to rAIns (Figure 4B). The rTPJ had the maximal positive (2.311) and the PCC had the maximal negative (−0.104) out-strength, whereas the MPFC had the maximal positive (1.237) and the rAIns had the maximal negative (−0.94) in-strength.

Contrasting the NEG group with the rest of the sample yielded increased self-inhibition of PCC and disinhibition of lDLPFC, and increased inhibitory input from lDLPFC to PCC and MPFC and from rTPJ to MPFC (Figure 4C). The PCC had the maximal positive (0.365) and the rTPJ and the lDLPFC had the maximal negative (−0.872) out-strength, whereas the rAIns had the maximal positive (0.43) and the MPFC had the maximal negative (−1.248) in-strength.

## Discussion

In this study, we investigated how self-appraisal is associated with brain activity using the multilevel mediation analysis of fMRI data obtained while the subjects performed the trait adjective judgment task. We proceeded from the assumption that the very process of self-appraisal should reflect the level of self-esteem, which could be indirectly measured by the strength of correlations between the emotional valence of presented adjective and its endorsement vs. rejection. Behavioral results seem to confirm this assumption. On average, this correlation was positive, in line with the notion that most people tend to evaluate themselves positively rather than negatively (Baumeister, 1999). The strength of this correlation was associated negatively with self-reported neuroticism and positively with conscientiousness, in line with the evidence linking trait self-esteem with these personality dimensions (Pullmann and Allik, 2000; Robins et al., 2001; Watson et al., 2002). Thus, this correlation could be considered a behavioral manifestation of positive self-bias, and in the further analysis, we investigated how this correlation is mediated by the brain. This analysis has revealed the rTPJ as the key region linking the emotional valence of the input stimuli with the behavioral response. Two other regions that also showed significant mediation effects included the left and right motor cortices. lPG activity correlated positively with both the adjective valence and the confirmatory response, whereas rPG showed negative associations with both these variables (see Figure 3B). Keeping in mind that the subjects responded “Yes” or “No” using their right and left hands, respectively, lPG mediation actually reflects the tendency to endorse positive self-descriptions, whereas rPG mediation reflects the tendency to reject negative ones. Thus, these two mediation effects could also be considered as a manifestation of positive self-bias. A number of other regions were significantly associated with different stages of task processing, although they did not show a significant mediation effect. Most of these regions coincide with areas that are consistently activated in self-appraisal tasks (e.g., MPFC, PCC, AIns, and DLPFC). Other regions (e.g., cuneus and lingual gyrus) are associated with more general functions, such as visual processing. We used the former regions along with the three regions, which showed significant mediation effects, as the nodes in the DCM analysis. This analysis also showed the prominent role of the rTPJ in causal interactions between the seven regions during the processing of adjective valence. Thus, in the whole sample of subjects, the rTPJ showed maximal positive out-strength, indicating that it is the primary driving force in the network.

The TPJ is a vaguely defined anatomical term labeling an area located between the temporal and parietal lobes. In terms of functional correlates, the left TPJ is mostly related to language and semantics processing (Binder et al., 2009), whereas the rTPJ is associated with a number of seemingly disparate processes ranging from spatial reorienting (Corbetta et al., 2000) to theory of mind (Saxe and Wexler, 2005). Bzdok et al. (2013), using multi-modal connectivity-based parcellation, revealed two distinct clusters within the rTPJ, with the anterior one being located around y = −39 and the posterior one around y = −54. The latter location nicely corresponds to the center of the rTPJ VOI (y = −52). In terms of functional characterization, the anterior cluster is associated with attentional processes and the posterior one with social cognition and memory retrieval (Bzdok et al., 2013). Activation within the posterior rTPJ is consistently documented in theory-of-mind and deception, as well as memory retrieval tasks (for reviews, see Saxe and Wexler, 2005; Saxe, 2006; Van Overwalle and Baetens, 2009); the topography of the posterior rTPJ network defined using connectivity analyses corresponds to meta-analytic definitions of the DMN (Bzdok et al., 2013). The leading role of the rTPJ in the process of self-appraisal, as revealed in this study, implies that self-evaluation is intimately linked with social cognition, supporting the existing theories of self-esteem as an interpretation of the opinion of others about oneself (Baumeister and Leary, 1995; Leary et al., 1995; Leary and Baumeister, 2000). Another possible explanation would be that in this task, the rTPJ is involved as a device for the retrieval of relevant memories and supplying them to frontal cortical regions associated with attention regulation and decision-making (i.e., the MPFC, DLPFC, and AIns). Indeed, DLPFC and AIns are the primary nodes of the central executive and salience networks (Seeley et al., 2007), whereas MPFC is involved in decision-making in the context of self-referential tasks (Gusnard et al., 2001). Interestingly, in the analysis of commonalities, MPFC showed maximal positive in-strength, meaning that it received maximal excitatory input from other regions. It also sent excitatory connections to main hubs of most networks (i.e., AIns, rTPJ, and PCC, see Table 2). It implies that the MPFC, along with the rTPJ, plays an essential role in the processing of adjective valence and in decision-making. The lDLPFC also sent excitatory connections to most other regions, such as PCC, rAIns, and both the left and right motor areas consistent with its function of the executive control center. rAIns, on the other hand, received the strongest inhibitory input (most notably from the PCC) and sent exclusively inhibitory connections to other regions (the strongest one being to the right motor cortex, see Table 2). If one mentally reverses the scores of the input variable (i.e., adjective valence), it becomes evident that with the increase in adjective negativeness rAIns increasingly receives and sends excitatory connections, which ultimately results in excitation of the right motor cortex (i.e., the readiness to say “NO”). This points out to a prominent role of the rAIns in the processing of negative self-descriptions as potentially harmful to the self-image, consistent with its involvement in harm avoidance (e.g., Paulus et al., 2003; Huggins et al., 2018). Summing up the results of the whole-sample mediation and DCM analyses, a prominent role of the two social brain regions, namely, the rTPJ and the MPFC, in the processing of adjective valence in the context of self-appraisal seems evident. rAIns seems to be involved in a defensive mechanism against negative self-descriptions that are potentially harmful to the self-image.

In line with the expectation, mediation analysis allowed to reveal individual differences in brain mechanisms underlying the two ways of self-esteem boosting. Noteworthy is that mediation effects were found to be consistent for the left and right motor areas, implying that the tendency to endorse positive self-descriptions and reject negative ones was consistently expressed in most of the participants. For the rTPJ, on the other hand, the mediation effect was found to be driven by a covariance between the *a* and *b* coefficients rather than by consistent effects. In approximately one-third of the sample, the increase in adjective valence was associated with increased rTPJ activation, which, in turn, was associated with an increased probability of a confirmatory response. In another third, on the contrary, the decrease in adjective valence was associated with rTPJ activation, which was associated with an increased probability of a negative response. The rest of the sample did not show consistent effects. Thus, it appears that for some individuals, the rejection of negative self-descriptions might be more important than the endorsement of positive ones. Independent sample *t*-test showed that on average representatives of the NEG group rejected negative self-descriptions more frequently than representatives of the POS group (*t* = 2.2, *p* = 0.037). Moreover, the number of rejected negative self-descriptions correlated strongly negatively with the reaction time in NEG (*r* = −0.841, *p* < 0.001) but not in the two other groups (both *p* > 0.3), meaning that the representatives of the NEG group who rejected more negative self-descriptions did it more promptly and without hesitation, which resembles an automatic defensive response.

In terms of psychological correlates, the POS group membership was predicted by lower scores on one of the behavioral activation facets measured by the drive scale of Carver and White and by lower Escore. The BAS scales of Carver and White have been constructed with an emphasis on positive emotionality. In the original study, the drive scale showed a substantial correlation with positive affectivity, as measured by PANAS (Carver and White, 1994). In other studies, it consistently showed correlations with different measures of extraversion, self-reported happiness, and reward reactivity (Jorm et al., 1998; Knyazev et al., 2004a; Smillie et al., 2006). In this sample, drive correlated positively with activity (a facet of extraversion) and negatively with inferiority (a facet of neuroticism). A lower Escore means a more negative evaluation of the person, which was selected as a figure of “Enemy.” In this sample, Escore correlated negatively with anger, hostility, and assertiveness. Therefore, in psychological terms, the POS group could be broadly characterized as individuals with higher scores on hostility and lower scores on reward-reactivity and positive emotionality. It appears that such a personality profile predisposes to the endorsement of positive self-descriptions, as mediated by the rTPJ. In the DCM analysis, contrasting POS with the rest of the sample showed increased self-inhibition of lDLPFC and rAIns and disinhibition of PCC, as well as a higher excitatory input from rTPJ to MPFC and PCC, and from MPFC to rTPJ, and a higher inhibitory input from MPFC to rAIns. Thus, in this group, relative to others, increased activation and communication between DMN hubs and inhibition of lDLPFC and rAIns is observed. The two latter regions are the main hubs of the so-called task-positive network (TPN), which is frequently contrasted with the DMN as being outward- and inward-oriented, respectively (Fox et al., 2005). The DMN and the TPN are frequently considered “anticorrelated networks,” meaning that activation of each one of them is usually accompanied by deactivation of the other (Fox et al., 2005; Allen et al., 2012; Chai et al., 2012). In resting condition, the “dominance” of the DMN over the TPN is associated with depressive symptomatology both in clinical and preclinical samples (Hamilton et al., 2011, 2013; Knyazev et al., 2016, 2018) and is interpreted as a reflection of increased self-focus (Hamilton et al., 2011; Menon, 2011). These findings are in line with observations showing that in non-clinical populations, mind wandering, which has been associated with DMN activity (Fox et al., 2015), is related to lower levels of happiness (Killingsworth and Gilbert, 2010). On the other hand, engaging in a demanding activity gives rise to the experience of “flow,” which is accompanied by deactivation of DMN and activation of TPN regions and a positive experience of pleasantness and intrinsic motivation (Csikszentmihalyi, 2000; Ulrich et al., 2016). One may speculate that in individuals with higher hostility and lower positive emotionality (i.e., the POS group), increased self-focus may be associated with “dominance” of the DMN over the TPN in the process of self-appraisal. In this group, the increase in adjective valence is associated with an increase in rTPJ activity and the excitatory input from rTPJ to the left motor cortex (see Figure 4B), which may serve as a mechanism for the endorsement of positive self-descriptions.

The NEG group membership was predicted by low anger. This group, relative to others, showed increased inhibition of the two major DMN hubs (the PCC and the MPFC) and disinhibition of the two TPN nodes. It seems reasonable to suggest that dealing with negative self-descriptions needs greater involvement of emotion processing and emotion regulation capacities, which is reflected in greater involvement of rAIns and lDLPFC (Schmitz and Johnson, 2007; Van der Meer et al., 2010; Brühl et al., 2014; Jiang et al., 2018). MPFC received maximal inhibitory input, whereas rAIns received maximal excitatory input and produced maximal inhibitory output. It appears that in this group, rAIns is generally more active than in the rest of the sample. Given the above-discussed role of this cortical area in defensive mechanisms, one may speculate that in this group, self-appraisal is mostly driven by the rejection of negative self-descriptions, which is secured by the inhibitory input from the rAIns to the left motor cortex (see Figure 4C).

The NONE group membership was predicted by low scores on fun-seeking. The fun seeking scale of Carver and White has been shown to measure mostly trait impulsivity (Smillie et al., 2006). In this sample, fun-seeking correlated moderately positively with sociability, impulsivity, risk-taking, irresponsibility, and extraversion, and negatively with conscientiousness. One may suggest that in these individuals, lack of spontaneity made it difficult to make a decision, which may result in a lack of consistent strategy.

An advantage of the experimental design is that it included not only self-appraisal but also appraisal of other people. Interestingly, significant group-level mediation effects were revealed only in the self-appraisal condition but not in tasks related to other persons. It implies relative uniformity of brain mechanisms underlying the self-appraisal and a considerable between-subject variability in their activity during the evaluation of others. On the other hand, interleaving self-appraisal with an evaluation of other persons might have triggered comparisons of the self with others and thus forced activation of the “social brain” regions.

Summing up, in this study, we aimed to answer two questions that remained unanswered in the field of self-esteem research, i.e., brain underpinning of the positive self-bias and the two ways of self-enhancement (i.e., endorsement of positive self-descriptions and rejection of negative ones). We show that the strength of correlation between the emotional valence of the presented adjective and its endorsement vs. rejection could be treated as a measure of positive self-bias, which is mediated by rTPJ activity that acts as a driving force in the network of brain regions, which are consistently activated in self-appraisal tasks (MPFC, PCC, rAIns, and lDLPFC). MPFC, along with the rTPJ, also plays an essential role in this network receiving maximal excitatory input from other regions. rAIns, on the other hand, received the strongest inhibitory input and sent exclusively inhibitory connections to other regions pointing out to its role in the processing of negative self-descriptions. Analysis of individual differences in the effect of mediation shows that in some individuals, the rTPJ increases its activity along with the endorsement of positive self-descriptions, whereas in others, it increases its activity along with the rejection of negative ones. The former group is characterized by higher hostility and lower positive emotionality, whereas the latter group is characterized by lower aggressiveness. In the former group, increased activation and communication between DMN hubs and inhibition of TPN hubs are observed, implying an increased self-focus in the process of self-appraisal, which is presumably driven by the endorsement of positive self-descriptions. In the latter group, self-appraisal is mostly driven by the rejection of negative self-descriptions and is accompanied by an increased activity of the rAIns and inhibition of the two major DMN hubs (the PCC and the MPFC).

## Data Availability Statement

The raw data supporting the conclusions of this article will be made available by the authors, without undue reservation.

## Ethics Statement

The studies involving human participants were reviewed and approved by the Scientific Research Institute of Neurosciences and Medicine ethical committee. The patients/participants provided their written informed consent to participate in this study.

## Author Contributions

GK planned the study, performed statistical analyses, and wrote the initial draft of the manuscript. AS, AB, and PR participated in data collection. AS and AB designed the experiment. PR wrote programs for running the experiment. All participated in manuscript correction and approved the final version.

## Conflict of Interest

The authors declare that the research was conducted in the absence of any commercial or financial relationships that could be construed as a potential conflict of interest.
